# Uncovering everyday attention in the lab: front-viewed heads boost overt social orienting

**DOI:** 10.1186/s41235-025-00661-2

**Published:** 2025-08-01

**Authors:** Mario Dalmaso, Anna Lorenzoni, Giovanni Galfano, Marta Riva, Luigi Castelli

**Affiliations:** Department of Developmental and Social Psychology, University of Padova, Via Venezia 8, 35131 Padua, Italy

**Keywords:** Everyday social interactions, Social attention, Head turn, Eye contact, Social cognition

## Abstract

Social attention can be defined as the tendency to orient attentional resources in response to spatial cues provided by others, such as their gaze or head direction. This mechanism is essential for navigating real-world environments, where rapidly and accurately interpreting others’ behaviour is often critical. Regarding head-driven orienting, research studies suggest that social attention can be enhanced when a front-facing head cue establishes eye contact (vs. no eye contact) with the observer, but also when the head cue is viewed from behind (vs. from the front), and hence, eye contact cannot be established. Across three experiments, we directly compared these two scenarios—which are highly common in everyday life—by presenting a central head cue showing either the front (establishing eye contact) or back, followed by a turn to the left or right. In Experiments 1 and 2, participants were required to manually respond to peripheral targets while ignoring the head cue, whereas in Experiment 3, oculomotor responses were recorded. Although the initial view of the head did not affect manual responses, eye movement data revealed enhanced social attention when the head was initially viewed from the front. These results suggest that eye movements provide a sensitive measure for detecting potential social modulations of attention. Moreover, eye contact confirms here its role as a powerful social signal for humans, capable of boosting overt orienting responses. Future research should explore these effects in more dynamic and ecologically valid settings, such as real social interactions.

## Introduction

Many studies have shown that averted gaze and head turns can trigger attentional shifts in an observer (e.g., Capozzi & Ristic, 2018; Frischen et al., 2007; Hietanen, 1999, 2002; Langton et al., 2000; McKay et al., 2021). This phenomenon, often labelled social attention,^1^ is crucial for social behaviour, as it allows people to focus on the same object during conversation or notice relevant events in the environment. A classic paradigm to study social attention involves presenting a central head that suddenly moves its eyes or turns to the left or right. After a given time interval (i.e., Stimulus Onset Asynchrony, SOA), a peripheral target appears either in the same location indicated by the social cue (a spatially congruent trial) or a different location (a spatially incongruent trial). Responses are typically faster and more accurate on congruent than incongruent trials, suggesting that the social cue was effective in reliably pushing attention (e.g., Driver et al., 1999; Friesen & Kingstone, 1998; Langton & Bruce, 1999).

Recent research has demonstrated that social attention is influenced by various factors related to facial cues (for a review, see Dalmaso et al., 2020b). Of particular interest for the current work, it has been shown that eye contact, a key social signal indicating approach behaviour, can enhance social attention (e.g., Hamilton, 2016). For instance, when an individual makes eye contact with an observer before indicating a direction (by either only moving the eyes or turning their head), the observer is more likely to shift their attention effectively than when no eye contact occurs (e.g., Xu et al., 2018). This could likely reflect the fact that eye contact tends to increase brain activity in areas such as the amygdala, and generally heightens physiological arousal (Hietanen, 2018). In turn, this increment in arousal would act as a booster for social attention. The enhancing effect of eye contact on attention orienting has been observed in studies where covert orienting of attention (i.e., without eye movements) was investigated. Participants manually responded to peripheral targets, with eye contact (vs. no eye contact) established with either human (Xu et al., 2018; for additional evidence, see also Bristow et al., 2007) or robotic faces (Kompatsiari et al., 2018, 2022). As an example, in Xu et al. (2018), participants were shown a face that could display either a direct gaze (eye contact condition) or a downward gaze (no eye contact condition). The face then looked left or right before a target appeared in a peripheral location, requiring a manual key press. Responses were faster on congruent than on incongruent trials and this difference was larger in the eye contact condition compared to the no eye contact condition. However, a similar study by Ishikawa et al. (2021) did not replicate this effect, likely because each trial started with an affective prime (i.e., a neutral or a threatening picture), which could have masked the impact of eye contact on social orienting. Another recent work (Dalmaso et al., 2020a) focused on overt orienting of attention and recorded eye movements. Participants were asked to perform saccades based on a directional ‘go’ signal, while a task-irrelevant face was initially displayed with either a direct gaze (eye contact) or an upward gaze (no eye contact) before gazing left or right. While the analyses of all saccades confirmed the presence of gaze-mediated attention shifts, these were not influenced by the eye contact condition. However, on incongruent trials, reflexive saccades—those erroneously directed toward the location indicated by the gaze stimulus—had shorter latencies than the voluntary saccades correctly executed in accordance with the 'go' signal. This difference emerged only after eye contact, thus suggesting that it can influence attention provided that sensitive measures are utilised. Importantly, it is worth noting that a conceptually similar effect has also been observed among infants, who have been shown to follow the gaze of adults only when it is preceded by ostensive communicative signals, such as eye contact (Senju & Csibra, 2008). This could be interpreted as further evidence for the key role of eye contact in social orienting.

In contrast to studies highlighting the importance of eye contact in social attention, Gallup et al. (2012a) found that observers were more likely to follow a person's gaze when viewing their head from behind rather than from the front. In this naturalistic study, pedestrians on a city street, unaware they were part of an experiment, followed the gaze of someone walking ahead of them (head from behind) more frequently than someone facing them. Gallup and colleagues offered possible explanations for this result. One relates to social norms, suggesting that individuals in naturalistic and (potentially) interactive settings often avoid fixating on the eye region of strangers (thus avoiding potential eye contact) because it may be perceived as inappropriate. Another one suggests that following the gaze of faces seen from the front may be less common, as it could diminish the natural tendency for people to engage in social interactions (see also Laidlaw et al., 2011). A third explanation is that individuals walking in the same direction as the observer are engaging with an environment that the observer will soon encounter, thereby making their spatial cues more relevant. Although Gallup et al. (2012a) did not measure eye contact directly, these findings suggest that—at least under certain conditions—the social attention system may be more sensitive to head cues even when the eyes are not visible, thus challenging the notion that eye contact is always associated with increased social attention (see also Gallup et al., 2012b).

The current work aimed to examine social attention responses to heads viewed from the front or back in well-controlled experimental settings, directly comparing these two stimuli. According to two opposite hypotheses, an increased orienting could be expected in response to heads initially viewed either from the front and establishing eye contact (e.g., Dalmaso et al., 2020a; Xu et al., 2018), or from the back (Gallup et al., 2012a). Across three experiments, we examined both covert orienting (where participants provided manual responses to discriminate peripheral targets; Experiments 1 and 2) and overt orienting (where saccadic eye movements were made in response to a central 'go' signal; Experiment 3). This approach was motivated by the idea that manual and oculomotor responses may reflect partially distinct stages of attentional processing. In particular, manual responses are typically slower and may incorporate more voluntary and/or strategic components, whereas eye movements generally provide a more rapid and direct measure of spatial orienting, often driven by reflexive mechanisms (e.g., Hutton, 2008). By assessing both response types, we aimed to determine whether the social cues we used could exert similar effects (or not) within the two types of measures (see also, e.g., Pereira et al., 2022). In all experiments, a central, task-irrelevant, head stimulus was centrally presented. On each trial, the head was initially presented from the front, establishing eye contact, or from the back, preventing the perception of the eyes. We anticipate here that, while manual responses were unaffected by the initial view of the head, saccadic data revealed stronger orienting when the head was seen from the front. This aligns with the notion that eye contact, rather than viewing the back of the head, can play a key role in boosting social attention (see also Dalmaso et al., 2020a; Kompatsiari et al., 2018, 2022; Xu et al., 2018).

## Methods

### Experiment 1: covert orienting of attention and one SOA

In this experiment, we designed a manual response task where a task-irrelevant central head was initially presented in either a front or back view. Then, the head turned left or right, and subsequently a peripheral target appeared. As a first step, we decided to use only a fixed SOA of 200 ms, in keeping with previous studies showing this duration to be optimal for detecting the effects of various social manipulations on gaze-mediated orienting (see also Dalmaso et al., 2020b).

### Participants

The sample size was calculated a priori based on guidelines for linear mixed-effects models (Brysbaert & Stevens, 2018; see also the results section). We decided to use linear mixed-effects models as they can account for subject-level variability. In short, a minimum of 1600 data points was required for each experimental condition. Given that the experimental design (see next section) allowed for 48 data points per cell, the minimum number of participants needed was 34. We decided to contact more participants to increase statistical power and account for potential participant withdrawal. Online data collection was stopped after approximately one week when no additional data were received. The final sample consisted of 47 students at the University of Padova (*Mean age* = 24 years, *SD* = 7.43, 7 males), all of whom participated voluntarily. The study was approved by the Ethics Committee for Psychological Research at the University of Padova (protocol number 4654).

### Stimuli, apparatus, and procedure

Head stimuli (width: 467 px; height: 500 px) were selected from Shutterstock (https://www.shutterstock.com). One male and one female actor were selected. For each identity, there were four different images: a front view of the face gazing ahead, the back of the head, a leftward-oriented head, and a rightward-oriented head. The experiment was programmed in PsychoPy and delivered online using Pavlovia. The screen background was set to white.

**Fig. 1:**
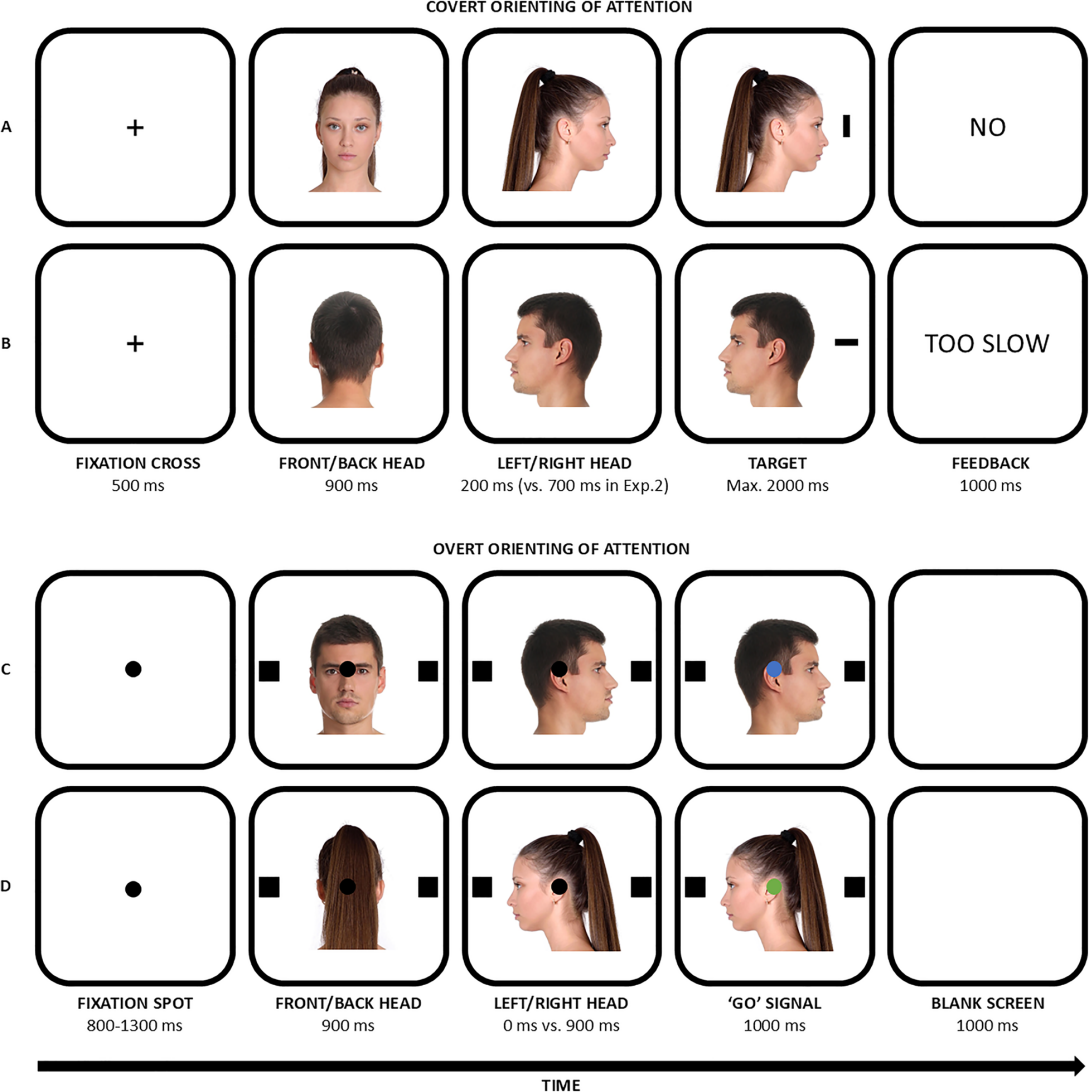
Illustration of the stimuli (not to scale) and trial sequences used in the three experiments. Panels A and B correspond to Experiments 1 and 2; Panels C and D refer to Experiment 3. The head cues depicted here were associated with whole-body rotations rather than ‘standard’ head turns, which typically involve smaller angular rotations. This choice ensured that the perceptual change was equivalent between front- and back-viewed heads

Each trial began with a central black fixation cross (Arial font, 0.1 normalised units) displayed for 500 ms, followed by a central image of a head for 900 ms, which could be shown in either a front or back view (see also Fig. 1). The same head stimulus then appeared, rotated left or right, for 200 ms (i.e., SOA). Finally, a black target line (width: 40 px; height: 12 px) appeared ± 0.8 normalised units to the left or right of the screen (calculated from the centre). Participants were instructed to use their left and right fingers to discriminate the orientation of the target line (vertical or horizontal) by pressing either the ‘f’ or ‘k’ key (counterbalanced across participants).^2^ They were told to ignore the head orientation, as it was non-informative of the target location, and to respond as quickly and accurately as possible. A trial ended when a response was given or after 2000 ms, whichever occurred first. Missing and incorrect responses were signalled with visual feedback (‘Too slow’ or ‘Error,’ respectively; Arial font, 0.05 normalised units) lasting 1 s. Participants completed a practice block of 10 trials, followed by three experimental blocks of 64 trials each (192 experimental trials in total). Each experimental condition appeared an equal number of times in random order.

## Results

Data were analysed in R. Missing responses were rare (0.86% of trials) and were discarded without further analysis. Incorrect responses (3.71% of trials) were discarded and analysed separately. Trials with correct responses that had latencies shorter than 150 ms or longer than 1500 ms (0.31% of trials) were considered outliers (for a similar approach, see also, e.g., Dalmaso et al., 2023a, 2023b, 2025) and excluded from further analyses. The experimental factors considered in the analyses were congruency (2 levels: congruent vs. incongruent) and initial head view (2 levels: front vs. back).

Latencies for correctly-responded trials were analysed using linear mixed-effects models of increasing complexity. The best-fitting model included congruency as fixed effect and participant as a random effect. The main effect of congruency was significant, *F*(1, 8535.1) = 9.066, *p* = 0.003, indicating that latencies were shorter on congruent trials (*M* = 596 ms, *SE* = 11.8) compared to incongruent trials (*M* = 605 ms, *SE* = 11.8).

For completeness, accuracy was analysed using linear mixed-effects logit models. The best-fitting model included congruency as a fixed effect and participant as a random effect. The main effect of congruency was significant (*b* =  − 0.234, *SE* = 0.111, *z* =  − 2.102, *p* = 0.036), showing that accuracy was higher on congruent trials (*M* = 0.97, *SE* = 0.003) than on incongruent trials (*M* = 0.96, *SE* = 0.004; see also Table 1).

**Table 1:** Mean RTs (in ms) and accuracy (proportion correct) as a function of SOA, initial head view, and congruency in Experiments 1–3. Note that in Experiment 1, only a single SOA (200 ms) was used, while in Experiment 2, the shorter SOA was 200 ms and the longer SOA was 700 ms. In Experiment 3, the shorter SOA was 0 ms and the longer SOA was 900 ms. The data reported here have been extracted from saturated models

	Shorter SOA				Longer SOA			
	Front		Back		Front		Back	
	Congruent	Incongruent	Congruent	Incongruent	Congruent	Incongruent	Congruent	Incongruent
*Experiment 1*								
*RTs*	594 (12)	604 (12)	598 (12)	605 (12)				
*Accuracy*	0.969 (0.004)	0.964 (0.005)	0.974 (0.004)	0.965 (0.005)				
*Experiment 2*								
*RTs*	645 (8.45)	654 (8.46)	652 (8.45)	659 (8.46)	589 (8.46)	599 (8.45)	586 (8.45)	605 (8.45)
*Accuracy*	0.970 (0.004)	0.962 (0.004)	0.969 (0.004)	0.962 (0.004)	0.964 (0.004)	0.970 (0.004)	0.967 (0.004)	0.971 (0.003)
*Experiment 3*								
*RTs*	399 (6.22)	418 (6.23)	405 (6.22)	422 (6.24)	329 (6.22)	332 (6.22)	328 (6.22)	332 (6.22)
*Accuracy*	0.961 (0.005)	0.915 (0.009)	0.973 (0.004)	0.903 (0.01)	0.952 (0.006)	0.942 (0.007)	0.949 (0.006)	0.947 (0.006)

## Discussion

Both the latency and accuracy analyses showed that the only reliable result was the main effect of congruency, indicating that head direction triggered covert orienting responses in the observers. This finding is consistent with previous studies (e.g., Dalmaso et al., 2023b; Han & Eckstein, 2023; Langton & Bruce, 1999; Langton et al., 2000; Lu & van Zoest, 2023; Visser & Roberts, 2018). The presentation of the head in either a front or back view did not affect the congruency effect, meaning that none of our two initial hypotheses—an increased orienting response to head stimuli initially viewed from either the front or the back—was confirmed. In the next experiment, we used a longer SOA to elucidate whether a modulation of eye contact might occur later in time. In this regard, previous studies reporting enhanced social attention after eye contact used a range of SOAs, all longer than the 200-ms SOA employed here (e.g., 500 ms and 1000 ms in Kompatsiari et al., 2018, 2022; and 300 ms and 529 ms in Xu et al., 2018). Therefore, in Experiment 2, we introduced an additional SOA of 700 ms to explore whether differences between front and back view stimuli would emerge later. This duration was chosen as a compromise based on previous research and because the 200–700 ms range has been shown to cover the optimal SOAs for detecting reliable gaze-mediated orienting of attention (McKay et al., 2021).

### Experiment 2: Covert orienting of attention and two SOAs

#### Participants

The sample size was calculated similarly to Experiment 1. Since the experimental design (see next section) allowed for 24 data points per cell, the minimum number of participants required was 67. We decided to contact more participants to increase statistical power and account for potential participant withdrawal. Online data collection was stopped after approximately one week when no additional data were received on the server. The final sample consisted of 114 new students at the University of Padova (*Mean age* = 23 years, *SD* = 6.81, 23 males), all of whom participated voluntarily. The study was approved by the Ethics Committee for Psychological Research at the University of Padova (protocol number 4654).

#### Stimuli, apparatus, and procedure

Everything was identical to Experiment 1, the only difference being the addition of a 700-ms SOA condition.

## Results

Data were analysed as in Experiment 1. Missing responses were rare (0.27% of trials) and were discarded without further analysis. Incorrect responses (4.11% of trials) were discarded and analysed separately. Trials with correct responses that had latencies shorter than 150 ms or longer than 1500 ms (0.46% of trials) were considered outliers and excluded from further analyses.

As for latencies, the best-fitting model included congruency, SOA, and head view (and their interactions) as fixed effects and participant as a random effect. The main effect of congruency was significant, *F*(1, 20,709) = 30.138, *p* < 0.001, indicating shorter latencies on congruent trials (*M* = 618 ms, *SE* = 8.09) compared to incongruent trials (*M* = 629 ms, *SE* = 8.09). The main effect of SOA was also significant, *F*(1, 20,709) = 825.2877, *p* < 0.001, with shorter latencies at the 700-ms SOA (*M* = 595 ms, *SE* = 8.09) compared to the 200-ms SOA (*M* = 653 ms, *SE* = 8.09). The main effect of head view was non-significant (*p* = 0.072), as well as all other results (*p*s > 0.116).

As for accuracy, the best-fitting model included congruency, SOA, and their interaction as fixed effects, with participant as a random effect. The main effect of congruency was significant, *b* =  − 0.235, *SE* = 0.095, *z* =  − 2.463, *p* = .014, indicating higher accuracy on congruent trials (*M* = 0.968, *SE* = 0.003) compared to incongruent trials (*M* = 0.966, *SE* = 0.003). The main effect of SOA was not significant (*p* = 0.148). However, the interaction between congruency and SOA was significant, *b* =  − 0.395, *SE* = 0.136, *z* = 2.892, *p* = .004, showing that the difference between congruent and incongruent trials was significant at the 200-ms SOA (*p* = 0.014) but not at the 700-ms SOA (*p* = 0.101; see also Table 1).

## Discussion

The results were consistent with the pattern observed in Experiment 1. The main effect of congruency was significant, confirming that head cues can orient covert attention (e.g., Langton & Bruce, 1999; Langton et al., 2000), as well as the main effect of SOA, aligning with a foreperiod effect. Importantly, the presentation of head stimuli in either front or back view did not influence the congruency effect. Therefore, the results from the first two experiments do not provide evidence in support of any of our initial hypotheses. In the next experiment, we used an oculomotor task, with two SOAs, similar to that employed by Dalmaso et al. (2020a), to investigate the impact of front/back head view manipulation on eye movements, including the comparison between reflexive and voluntary saccades. We moved to an oculomotor task because saccadic measures are strongly modulated by the nature of social stimuli (e.g., Mirabella et al., 2024) and have been shown to be more sensitive than manual responses for capturing spatial attention effects (e.g., Pereira et al., 2022).

### Experiment 3: Overt orienting of attention and two SOAs

#### Participants

The sample size was calculated as in the previous two experiments. Since the experimental design (see the next section) allowed for 28 data points per cell, the minimum required number of participants was approximately 58. We decided to test more participants to increase statistical power and account for the fact that not all participants show reflexive saccades (see, e.g., Dalmaso et al., 2020a). Laboratory-based data collection was stopped at the end of pre-scheduled booking sessions. The final sample consisted of 91 new students at the University of Padova (*Mean age* = 21 years, *SD* = 3.36, 18 males), all of whom participated voluntarily. The study was approved by the Ethics Committee for Psychological Research at the University of Padova (protocol number 4654).

#### Stimuli, apparatus, and procedure

The facial stimuli were the same as those used in the previous two experiments. Eye movements were recorded from the right eye using an EyeLink 1000 Plus (SR Research Ltd, Ottawa, Canada) at a frequency of 1000 Hz. Participants were seated 70 cm from a 24-inch monitor (1280 × 1024 pixels, 120 Hz), with a chinrest used to prevent head movements. A display PC running Experiment Builder (https://www.sr-research.com) controlled the timing and presentation of the stimuli. The screen background was set to white.

The task, based on the oculomotor interference paradigm devised by Ricciardelli et al. (2002), was conceptually similar to the previous two experiments but adapted for oculomotor responses (see also Fig. 1). First, participants completed a calibration and validation process, after which the experiment began. Each trial started with a central black fixation spot (diameter: 0.5°), flanked by two black squares as placeholders (side: 0.9°), positioned 9.7° to the left and right of the fixation spot. A 500-Hz tone, lasting 100 ms, signalled the start of the trial. Participants focused on the central spot, and the trial only continued if they maintained their gaze on the spot for a variable duration (ranging from 800 to 1300 ms, in 100 ms steps), monitored by a gaze-contingent trigger (invisible boundary diameter: 4°). If participants failed to maintain fixation within a 10-s period, a visual feedback (the word ‘Recalibration’) appeared for 2000 ms, the trial was aborted and recycled at the end of the block, and a new calibration and validation process was conducted.

If fixation was successful, a head stimulus appeared in the centre of the screen for 900 ms, followed by the same head stimulus rotated left or right. After either 0 ms (simultaneously with the presentation of the rotated head) or 900 ms (SOA), the central spot turned green or blue, indicating the direction of the saccade and serving as the 'go' signal. These two SOAs (different from the previous two experiments) were specifically chosen to adapt to the oculomotor nature of this task and tapping into more exogenous (i.e., it is known that the interference effects are at their maximum when the ‘go’ signal and the gaze stimulus appear simultaneously) and endogenous processes, according to previous studies using a similar oculomotor task (see, e.g., Dalmaso et al., 2020a; Kuhn & Kingstone, 2009). In addition, previous works reported a modulatory role of social variables on saccadic eye movements specifically at the 0-ms SOA (e.g., Zhang et al., 2021), including the boosting effect of eye contact (see Dalmaso et al., 2020a). Upon noticing the colour change, participants had to make a saccade to either the left or right placeholder. Half of the participants associated the green colour with a leftward saccade and the blue colour with a rightward saccade, while for the other half, the association was reversed. Participants were instructed to ignore the head stimulus, as its direction was not predictive of the required saccade direction. They had a maximum of 1000 ms to perform the saccade. Afterwards, a blank screen appeared for 1000 ms, during which participants were instructed to return their gaze to the centre before the next trial started.

There was a practice block consisting of 10 trials, followed by four experimental blocks of 56 trials each (224 experimental trials in total). Each experimental condition appeared an equal number of times in random order. A drift check was performed before each block.

## Results

Eye movements with a speed over 30°/s, acceleration over 8000°/s^2^, and an amplitude greater than one degree were classified as saccades (for a similar approach, see also, e.g., Kuhn & Kingstone, 2009). For each trial, we recorded the first, initial, saccadic eye movement without a blink that occurred after the ‘go’ signal. Saccades moving in the same direction as the ‘go’ signal were marked as correct, while those moving in the opposite direction were marked as wrong (7.25% of trials). The latency of correct saccades was the time from the ‘go’ signal to the start of the saccade. Correct saccades with a latency smaller than 80 ms or greater than 800 ms (0.99% of trials) were considered outliers (for a similar approach, see also, e.g., Dalmaso et al., 2023a) and discarded from further analyses.

Latencies and accuracy were analysed as in the previous two experiments. As for latencies, the best model fitting the data included congruency, SOA, initial head view, and their interactions as fixed effects and participant as a random effect. The main effect of congruency was significant, *F*(1, 18,224) = 60.616, *p* < 0.001, showing that latencies were shorter on congruent trials (*M* = 365 ms, *SE* = 6.04) compared to incongruent trials (*M* = 376 ms, *SE* = 6.05). The main effect of SOA was also significant, *F*(1, 18,226) = 4446.040, *p* < 0.001, showing that latencies were shorter at the 900-ms SOA (*M* = 330, *SE* = 6.04) compared to the 0-ms SOA (*M* = 411, *SE* = 6.05). The main effect of initial head view was also significant, *F*(1, 18,224) = 4.354, *p* = 0.037, showing that latencies were shorter for front view (*M* = 369, *SE* = 6.04) compared to the back view (*M* = 372, *SE* = 6.05). The interaction between congruency and SOA was significant, *F*(1, 18,224) = 34.000, *p* < 0.001, indicating that the difference between congruent and incongruent trials was significant at both SOAs (*ps* < 0.028), but the difference was greater at the shorter SOA (18 ms vs. 4 ms). The interaction between SOA and initial head view was also significant, *F*(1, 18,224) = 5.515, *p* = 0.019, but not further analysed due to its limited theoretical relevance (i.e., the congruency factor, essential to assess the magnitude of social attention, was not involved). The interaction between congruency and initial head view, as well as the three-way interaction, were both non-significant (*ps* > 0.732).

As for accuracy, the model best fitting the data included congruency, SOA, and their interaction as fixed effects, with participant as a random effect. The main effect of congruency was significant, *b* =  − 1.076, *SE* = 0.088, *z* =  − 12.219, *p* < .001, showing that accuracy was higher on congruent trials (*M* = 0.960, *SE* = 0.004) compared to incongruent trials (*M* = 0.929, *SE* = 0.006). The main effect of SOA was also significant, *b* =  − 0.432, *SE* = 0.096, *z* =  − 4.494, *p* < .001, showing that accuracy was higher at the 900-ms SOA (*M* = 0.948, *SE* = 0.005) compared to the 0-ms SOA (*M* = 0.945, *SE* = 0.005). The congruency × SOA interaction was also significant, *b* = 0.965, *SE* = 0.122, *z* = 7.876, *p* < .001, indicating that the difference between congruent and incongruent trials was significant at the 0-ms SOA (*p* < 0.001) but not at the 900-ms SOA (*p* = 0.192; see Table 1).

**Fig. 2:**
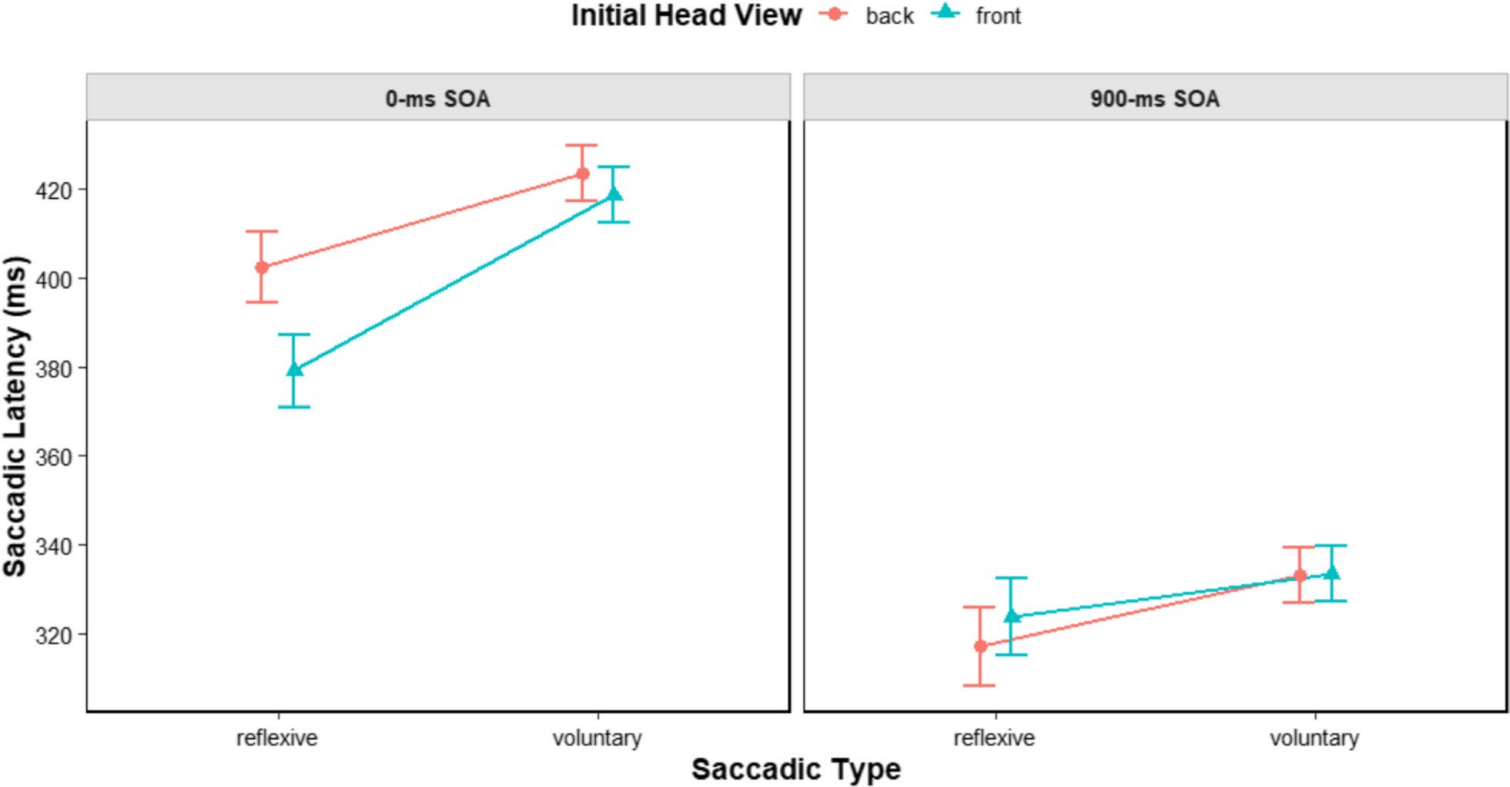
Saccadic latencies for reflexive and voluntary saccades as a function of initial head view and SOA. Error bars represent the standard error of the mean

Finally, we analysed reflexive (867 trials) and voluntary (8981 trials) saccades extracted from incongruent trials (for a similar approach, see also, e.g., Kuhn & Benson, 2007; Kuhn et al., 2010). It should be noted that on incongruent trials only, the head cue was associated with a given direction (e.g., left) and the ‘go’ signal with the opposite one (in this example, right). So, reflexive saccades were those erroneously directed towards the location suggested by the head stimulus, whereas voluntary saccades were those correctly performed based on the direction conveyed by the ‘go’ signal. All participants were included in the analyses as they exhibited both types of saccades. We considered saccade type (2: reflexive vs. voluntary), initial head view, and SOA as factors. The best model fitting the data included saccade type, initial head view, SOA, and their interactions as fixed effects, with participant as a random effect. The main effect of saccade type was significant, *F*(1, 9796.6) = 17.222, *p* < 0.001, showing that latencies were shorter for reflexive saccades (*M* = 365 ms, *SE* = 6.04) compared to voluntary saccades (*M* = 376 ms, *SE* = 6.05). The main effect of SOA was also significant, *F*(1, 9750.3) = 2660.311, *p* < 0.001, showing that latencies were shorter at the 900-ms SOA (*M* = 327, *SE* = 6.43) compared to the 0-ms SOA (*M* = 406, *SE* = 6.29). The main effect of initial head view was non-significant (*p* = 0.077), as was the interaction between saccade type and initial head view (*p* = 0.125). The interactions between saccade type and SOA, and SOA and initial head view were both significant (*ps* < 0.036), and were further qualified by the three-way interaction, *F*(1, 9752.7) = 4.301, *p* = 0.038. The three-way interaction was further analysed by considering each level of SOA separately. At the 0-ms SOA, the two main effects (*ps* < 0.0012) and the two-way interaction were significant (*p* = 0.014). The interaction showed that the difference between reflexive and voluntary saccades was significant for both front- and back-viewed heads (*ps* < 0.001), but the magnitude was greater in the former case (43 ms vs. 24 ms). At the 900-ms SOA, all results were non-significant (*ps* > 0.161; see also Fig. 2).

## Discussion

The results of Experiment 3 confirmed reliable overt orienting in response to head orientation, as reflected in both saccadic latencies and accuracy. Additionally, saccadic latencies showed a main effect of the initial head view, with reaction times being shorter for front-viewed heads compared to back-viewed ones. This finding is consistent with previous studies in which direct-gaze stimuli were employed (e.g., Dalmaso et al., 2020c; Quadflieg et al., 2004) and may reflect the greater arousal elicited by eye contact, prompting quicker responses from participants (see also Hietanen et al., 2016). Another possibility is that the stimulus sequence we employed created a peculiar low-level visual transient that, in turn, enhanced attentional disengagement and guided saccades toward the peripheral target. Additional studies are needed to fully capture the nature of this result. The primary finding, however, was that the difference, on incongruent trials, between reflexive saccades (i.e., those made following the direction of head cues) and voluntary saccades (i.e., those made following the direction associated with the ‘go’ signal; see also, e.g., Kuhn & Benson, 2007; Kuhn et al., 2010) was larger for front-viewed heads than for back-viewed heads. This effect appeared only at the 0-ms SOA, likely because at the 900-ms SOA participants had enough time to exert more control over saccade preparation and execution (see also, e.g., Dalmaso et al., 2015, 2020a; Zhang et al., 2021). Overall, the analysis of reflexive and voluntary saccades serves as a conceptual replication of Dalmaso et al. (2020a), confirming that eye contact (vs. no eye contact) enhances the more reflexive components of social attention, as revealed by saccadic eye movement analyses. This pattern likely reflects a biologically grounded mechanism linking the processing of eye-gaze stimuli with the more reflexive components of saccadic eye movements, a possibility discussed in greater detail in the next section.

## General discussion

The literature on social attention suggests that a head stimulus viewed from the front, establishing eye contact with an observer, can enhance attentional orienting (e.g., Bristow et al., 2007; Dalmaso et al., 2020a; Xu et al., 2018). However, some evidence also indicates that social attention may increase when the cueing head is viewed from the back (a condition in which the eyes are not visible) rather than from the front (Gallup et al., 2012a, 2012b). This study aimed to directly compare these two possibilities through three experiments where task-irrelevant head cues were initially presented in either a front or back view and then rotated leftwards or rightwards.

In Experiments 1 and 2, participants made manual responses to discriminate peripheral targets, and we observed reliable covert attentional shifts based on head orientation. These results are consistent with previous research (e.g., Langton & Bruce, 1999; Langton et al., 2000), demonstrating that head cues effectively push attention. However, unlike other studies that used manual responses and reported modulatory effects of eye contact on attention (Bristow et al., 2007; Kompatsiari et al., 2018, 2022; Xu et al., 2018), our findings revealed no such modulation by the initial view of the head (front vs. back). Nevertheless, this aligns with the results from Ishikawa et al. (2021), who also found no effect of eye contact on attentional shifts when head cues were presented. Taken together, these results suggest that while head direction can influence covert attention, the anticipated enhancement from eye contact might not be as pervasive in manual response paradigms (see also Ishikawa et al., 2021).

Experiment 3 focused on saccadic eye movements recorded in response to a central ‘go’ signal. This experiment replicated the previous findings, demonstrating that the overall analysis of all saccades revealed reliable overt orienting behaviour, with participants showing better performance on congruent over incongruent trials (see also, e.g., Ciardo et al., 2014; Kuhn & Benson, 2007; Ricciardelli et al., 2002; Zhang et al., 2021), and a lack of a modulatory role of the initial view of the head. Nonetheless, the analysis of reflexive versus voluntary saccades revealed a more pronounced difference for head cues initially viewed from the front, suggesting that eye contact enhanced the reflexive components of social attention.^3^ This finding is consistent with results reported by Dalmaso et al. (2020a), where a similar effect was observed when participants were exposed to faces establishing eye contact versus those with averted gaze. Notably, the observed modulation of head orientation occurred at the 0-ms SOA, which may be interpreted as an early-rising impact of eye contact on social attention. This aligns with previous studies (Dalmaso et al., 2015, 2020a; Zhang et al., 2021) in which the role exerted by social modulators was visible only when SOA was sufficiently brief to shield exogenous processing from top-down control.

In the current context, the distinction between manual responses and eye movements appears to be crucial, and our findings emphasise the value of using oculomotor measures. As a first consideration, it is important to recall that eye movements provide a more direct and ecologically valid measure than manual responses, as they closely mimic how we naturally respond to spatial cues in everyday social interactions. In other words, eye movements represent the primary tool we use to scrutinise the surrounding physical and social environments to gain potentially relevant information. Manual responses, on the contrary, represent a more peripheral proxy resulting from a chain of processes that are not all directly related to the exploration of the environment. This, in turn, may introduce additional variability and, therefore, potentially mask subtle effects related to social orienting. Moreover, eye-tracking offers a rich dataset that can better capture social attentional mechanisms (e.g., Dalmaso, 2022; Dalmaso et al., 2017a, 2017b; Edwards et al., 2015; Kuhn et al., 2010; Pfeiffer et al., 2013; Yokoyama et al., 2012), due to the collection of continuous, high-resolution spatial and temporal data, which can also likely reduce measurement noise and increase statistical power, thereby facilitating the detection of fine-grained attentional modulations (see also, e.g., Bompas et al., 2017; Malienko et al., 2018). In addition, the fact that the modulatory role of eye contact emerged in the more reflexive component of eye movements discussed above may also be explained at a biological level. According to the ‘Fast track modulator’ model proposed by Senju and colleagues (Burra et al., 2019; Senju & Johnson, 2009), faces that establish eye contact with an observer are rapidly processed by a specific subcortical system, with the Superior Colliculus (SC) playing a central role. In turn, the SC is well-known for generating saccadic eye movements, especially those made more reflexively (e.g., Lee et al., 1988; Sereno et al., 2006). Therefore, the enhanced reflexive orienting observed in Experiment 3 could be attributed to increased SC activity, likely triggered by direct eye contact (for a similar reasoning, see also Dalmaso et al., 2020a).

While our results support the idea that eye contact (as opposed to viewing the back of the head) enhances social attention, it is important to acknowledge that the findings by Gallup et al., (2012a, 2012b) may reflect specific dynamics of real-life social interactions that are difficult to be fully captured in current human–computer experiments. Indeed, in everyday social contexts, interactions are governed by social norms that may not apply in a laboratory setting. Gallup and colleagues (2012a, b) suggested that decreased gaze-following in response to frontal cues could be explained by different factors. For instance, on the one hand, there is a tendency to avoid eye contact with strangers; on the other hand, individuals walking ahead and showing the back of their heads may be perceived as offering more relevant spatial information about an environment the observer is about to navigate. These factors are less applicable to human–computer interactions, where participants typically respond to static images on a monitor. For completeness, it is also worth noting that, in real-world settings, front-viewed faces can still serve as important social cues, particularly when they convey emotional expressions. In this regard, a work by the same group (Gallup et al., 2014) showed that pedestrians were more likely to follow the gaze of front-viewed individuals when these communicated fearful or ‘suspicious’, rather than neutral, or happy, emotions. This contrasts with many lab-based studies on social orienting, where emotional expressions have often yielded inconsistent or even null results (see Dalmaso et al., 2020b, for a review). In sum, future research could benefit from more immersive and interactive tasks, such as those employing videos, virtual reality or avatars (e.g., Colombatto et al., 2020; Nummenmaa et al., 2009; Pfeiffer et al., 2013; Schilbach et al., 2012), which may better simulate real-life social dynamics.

Another distinction between Gallup et al. (2012a, b) work and our study is the lack of information regarding eye contact in their pedestrian experiments. Unlike computer-based settings (such as ours), where participants are continually exposed to eye contact, real-life scenarios may involve less frequent eye contact, potentially accounting for the differences in results. This highlights the importance of context in shaping social attention. For this reason, future studies could also explore the use of portable eye-tracking technology to study the effects of eye contact in natural, real-world scenarios. Portable eye trackers allow researchers to capture gaze behaviour in dynamic and more ecologically valid environments, offering a unique opportunity to investigate how social attention functions during everyday interactions (see, e.g., Bianchi et al., 2020). This method would help bridge the gap between the more controlled computer-based experiments and real-life social interactions, providing deeper insights into how eye contact influences attention outside the lab.

The present results can also be discussed in the context of previous research, which addressed the existence, if any, of a hierarchy of prioritisation among different social signals for pushing spatial attention. According to a pioneering study conducted by Perrett et al. (1992), eye gaze would dominate over other social cues, such as head and body orientation. However, according to subsequent studies, these three cues would not operate in isolation but instead interact dynamically (e.g., Hietanen, 2002; Langton et al., 2000). While our study cannot disentangle the specific priority assigned to different social cues, given that eye gaze and head orientation always provided the same spatial vector, it does nevertheless contribute to shed light on the importance of the characteristics of the stimulus preceding the spatial cue.

In conclusion, our study provides evidence that eye contact can enhance social attention, supporting the broader notion that eye contact has significant implications for human behaviour. This finding aligns with previous research (Bristow et al., 2007; Dalmaso et al., 2020a; Kompatsiari et al., 2018, 2022; Xu et al., 2018) and further corroborates the implications of eye contact for social cognition and interpersonal interactions (e.g., Conty et al., 2016; Dalmaso et al., 2022; Hamilton, 2016; Hietanen, 2018; Senju & Hasegawa, 2005). The increase in social attention was evident in oculomotor metrics, which may reflect the involvement of the SC, which is known to be implicated in both direct gaze processing and the generation of reflexive eye movements.

## Data Availability

Data, codes, and experiments can be found here: 10.17605/OSF.IO/5E3QW.

## Footnotes

1 Please note that the term social attention can refer to a broad range of attentional phenomena occurring in social contexts, such as the orienting of attention toward people. In the present work, however, we use this term specifically to refer to mechanisms involved in orienting attention in response to spatial social cues (e.g., head direction).

2 We opted for a discrimination task as it has been used in several studies exploring the impact of social variables on social attention (e.g., Bayliss et al., 2005; Jones et al., 2010), and to ensure greater consistency with the oculomotor task described in Experiment 3, where participants were asked to discriminate between two distinct ‘go’ signals.

3 We acknowledge that front- and back-viewed heads differ not only in terms of eye contact but also in overall perceptual complexity, as front-viewed heads display full facial features whereas back-viewed heads show only the hair (at least in our case). Future studies could attempt to isolate the specific impact of eye contact more precisely by including, for instance, a condition with downward gaze, representing a non-eye contact scenario.
